# The Biological Metallic versus Metallic Solution in Treating Periprosthetic Femoral Fractures: Outcome Assessment

**DOI:** 10.1155/2016/2918735

**Published:** 2016-11-20

**Authors:** Serafino Carta, Mattia Fortina, Alberto Riva, Luigi Meccariello, Enrico Manzi, Antonio Di Giovanni, Paolo Ferrata

**Affiliations:** Department of Medical and Surgical Sciences and Neuroscience, Section of Orthopedics and Traumatology, University of Siena, University Hospital “Santa Maria alle Scotte”, Siena, Italy

## Abstract

*Introduction*. The periprosthetic fracture of the femur is, in order of frequency, the fourth leading cause (5.9%) of surgical revision. Our study aims to demonstrate how the grafting of bone splint betters the outcomes.* Materials*. We treated 15 periprosthetic femoral fractures divided into two groups: PS composed of 8 patients treated with plates and splints and PSS involving 7 patients treated only with plates. The evaluation criteria for the two groups during the clinical and radiological follow-up were the quality of life measured by the Short Form (36) Health Survey (SF-36), Harris Hip Score (HHS), Modified Cincinnati Rating System Questionnaire (MCRSQ), bone healing measured by the Radiographic Union Score (RUS), postoperative complications, and mortality. The evaluation endpoint was set at 24 months for both groups (*p* < 0.05).* Results*. The surgery lasted an average of 124.5 minutes for the PS group and 112.6 minutes for the PSS. At 24 months all clinical and radiographic scores were *p* < 0.05 for the PS group. During follow-up 4 patients (2 in each group) died of causes not related to surgery.* Conclusions*. The use of the metal plate as opposed to cortical allogenic splint should be taken into consideration as a noteworthy point for periprosthetic femoral fractures.

## 1. Introduction

Fractures around the hip joints are commonly defined as periprosthetic fractures in opposition to specific fractures of the prosthetic components whose treatment requires the total or partial removal of the implants and their replacement. The number of orthopedic hip and knee implants is progressively increasing, due to the aging of the population. The highest number of complications in total arthroprosthesis is represented by the loosening and osteolysis. The degree of osteolysis increases over the time for the stress exercised on the implants and the progressive age-related structural changes of the bone leading to fractures [1, 2]. From a Mayo Clinic study in the US we may see that the incidence of supracondylar fractures after total knee replacement is 0.6–2.5%. Such fractures may occur more than 10 years after the first implant. However the incidence of periprosthetic hip fractures after the first implant was 1.1% and increased to 4% after the complete revision [2]. The implant may impair healing of the fracture due to endosteal ischemia [3]. Percentages of nonunion for proximal supracondylar fractures of total knee prosthesis are higher than those for fractures. The surgeon must restore the biomechanical integrity of the bone. This requires the restoration of a biological environment in which the bone can heal completely and resume its stability and support functions [4–9]. The treatment must include the soft tissue preservation in order to preserve the periosteal and/or endosteal blood supply. The surgeon should minimize the periosteal damage and consider the possibility of using bone grafts if the biological environment is compromised [4–9]. The patient's condition should be optimized. The goals of treatment are early functional recovery, in order to prevent pulmonary complications, bedsores, osteoporosis from disuse, and all the other complications of a prolonged bed rest, and the restoration of the axial alignment to help prevent the eccentric stress on the prosthesis and promote stabilization and early mobilization of the limb and prevent stiffness and muscle atrophy. The goals of treatment must be [4–9]

1. restoring the best possible anatomical axis;
2. obtaining the stability of both the prosthetic implant and the fracture;
3. obtaining early patient mobilization;
4. possibly guarantying a return to the quality of life before the trauma.

Modern conservation of cancellous or cortical bone matrix technologies allow in orthopedic surgery the use, as an aid, of biological materials of bone grafts to recreate a successful recovery of the medial wall and then bring the femoral structure to bear, once again, preinjury mechanical loads. Moreover, many authors and biomechanical studies on cadavers have shown that the medial splint is an indispensable counterfort to the medial plate in the osteosynthesis of periprosthetic hip and knee fractures. The purpose of our study is to demonstrate how the grafting of bone splint associated with the best metal plate improves fracture consolidation, functional recovery, and quality of life, compared to internal fixation with a simple plate [10–12].

## 2. Materials and Methods

From January 2010 to December 2014, at the UOC Orthopedics and Traumatology University of the AOUS Policlinico Santa Maria alle Scotte of Siena, we treated 15 diaphyseal periprosthetic femur, hip, and knee fractures. We divided the patients into two groups.

All patients were informed in a clear and comprehensive way of the two types of treatment and other possible surgical and conservative alternatives. Patients were treated according to the ethical standards of the Helsinki Declaration and were invited to read, understand, and sign the informed consent form.

The two patient groups were formed based on the patient's choice to undergo such treatment. Exclusion criteria included fractures caused by hematological or oncological pathologies, the age being less than 65, and patients who did not adhere to a minimum follow-up of 24 months.

The first group (PS) was represented by 8 patients treated with plate, ring, screws, and bone splint and bone grafts for the hip or knee periprosthetic fracture. The population of the PS group at the time of the trauma had a mean age of 77.8 years (range 70–89); the relation between the sexes (m : f) was 0.6 (3 : 5). The fractures were classified according to the Vancouver classification for the periprosthetic fracture of the hip and that of Rorabeck for the knee and they were so divided: Vancouver B2: 2; Vancouver C: 3; Rorabeck Type 2: 2; Rorabeck Type 3: 1 (Table 1). All patients underwent presurgery anesthetic visit. The preoperative risk for the most part was based on the ASA physical status classification system: III in 6 cases; 5 (62.5%) patients required a place in the intensive care unit for a postoperative recovery (Table 1). The most frequent comorbidities were the cardiovascular diseases which affected 75% (6 patients) of PS population and 62.5% (5 patients) of the patients had three or more comorbidities at the time of the trauma (for a more detailed description see Table 2). 50% of the patients (*n* = 4) were undergoing pharmacological treatment for osteoporosis at the time of the trauma. The average years of follow-up were 2.3 (range 1–4). All patients in the PS group were treated with bone from cadaveric bank that was implanted only after routine procedures according to protocol. In the immediate postoperative period, all patients followed a personalized physiotherapy program, according to their medical conditions.

The second group (PSS) was represented by 7 patients suffering from periprosthetic hip fractures treated exclusively with plate and screws while the knee (fractures) was treated with plate or revision prosthesis. The PS group population at the time of the trauma had an average age of 75.3 years (range 67–81); the ratio between sexes (m : f) was of 0.75 (3 : 4). Even in this group the fractures were classified according to the Vancouver classification for the periprosthetic fractures of the hip and that of Rorabeck for those of the knee and they were so divided: Vancouver B2: 2; Vancouver C: 3; Rorabeck Type 2: 2; Rorabeck Type 3: 1 (Table 1). All patients underwent presurgery anesthetic visit. The preoperative risk for the most part was based on the ASA physical status classification system: III in 4 cases; 5 (71.42%) patients required a place in the intensive care unit for a postoperative recovery (Table 1).

The most frequent comorbidity was the Diabetes Mellitus involving 85.71% (6 patients) of PS population and 71.42% (5 patients) of the patients had three or more comorbidities at the time of the trauma (for a more detailed description see Table 2). 85.71% of the patients (*n* = 6) were undergoing pharmacological treatment for osteoporosis at the time of the trauma. The average years of follow-up were 2.3 (range 1–5). The chosen criteria to evaluate the two groups during the clinical and radiological follow-up were the quality of life measured by the Short Form (36) Health Survey's (SF-36) overall score [13], the hip function and quality of life related to it, measured by the Harris Hip Score (HHS) [14], the knee function and quality of life related to it, measured by the Modified Cincinnati Rating System Questionnaire (MCRSQ) [15], the bone healing measured by Radiographic Union Score (RUS) [16], and postoperative complications.

The evaluation endpoint was set at 24 months for both groups.

## 3. Statistical Analysis

Descriptive statistics were used to summarize the characteristics of the study group and subgroups, including means and standard deviations of all continuous variables. The *t*-test was used to compare continuous outcomes. The Fisher's exact test (groups are smaller than 10 patients) was used to compare categorical variables. The statistical significance was defined as *p* < 0.05. We used Pearson correlation coefficient (*r*) to compare the predictive score of outcomes and quality of life. Statistical analyses were performed with SPSS v.15.0 (SPSS Inc., an IBM Company, Chicago, IL, USA). Mean ages (and their standard deviations) of the patients were rounded at the closest year. The predictive score of outcomes and quality of life and their standard deviations were approximated at the first decimal while Pearson correlation coefficient (*r*) was approximated at the second decimal.

## 4. Results

The surgery lasted an average of 124.5 minutes (92–186) for the PS group and 112.6 minutes in the PSS group (79 min–192 min).

**Figure 1:**
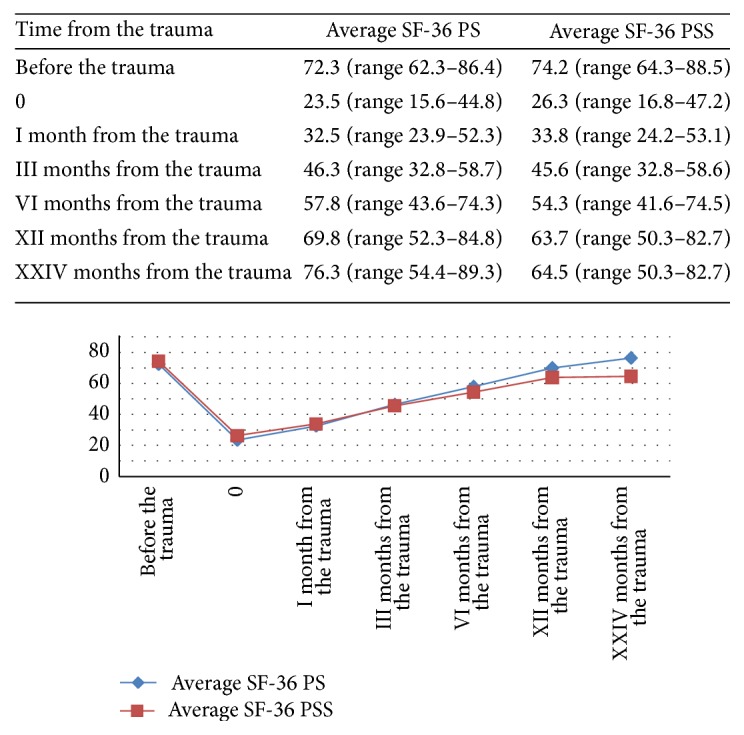
Trend of the follow-up to two years of quality of life measured by the Short Form (36) Health Survey (SF-36). At the sixth month of follow-up there was already a statistically significant difference (*p* < 0.05) in favor of the PS Group.

The quality of life before the trauma SF-36, for the PS group, was about 72.3 (range 62.3–86.4) points while that of the PSS group was 74.2 (range 64.3–88.5) points; there was no statistically significant difference between the two groups (*p* > 0.5). In the sixth month the SF-36 score was 57.8 (range 43.6–74.3) for the PS group, while that of PSS was 54.3 (range 41.6–74.5); there was a statistically significant difference (*p* < 0.05) in favor of the PS group. For a more detailed description see Figure 1.

**Figure 2:**
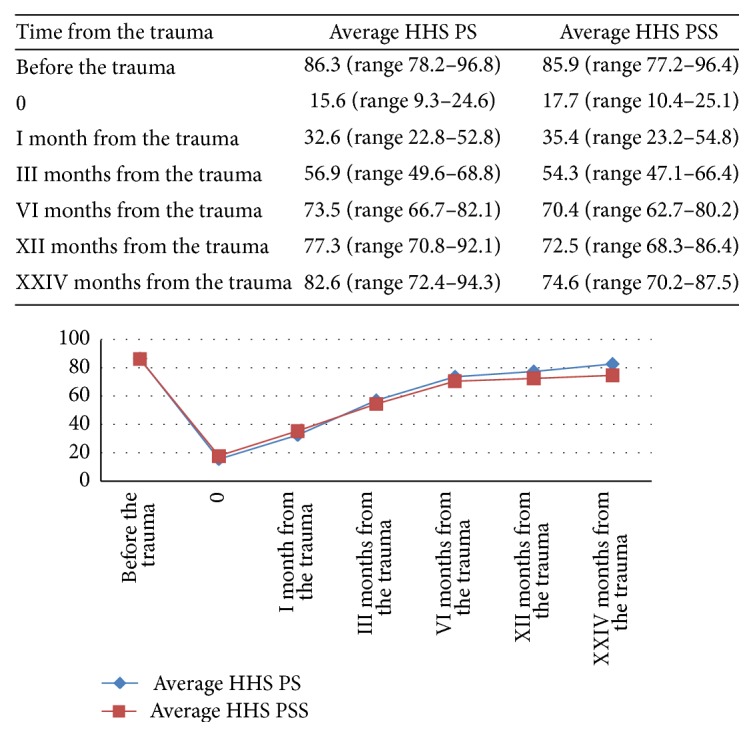
Trend hip function and quality of life related to it for 2-year follow-up measured by Harris Hip Score (HHS). At six months there was a statistically significant difference (*p* < 0.05) in favor of the PS group.

HHS before the trauma, for the PS group, was about 86.3 (range 78.2–96.8) points while that of the PSS group was 85.9 (range 77.2–96.4) points. There was no statistically significant difference between the two groups (*p* > 0.5). In the sixth month, the HHS score was 73.5 (range 66.7–82.1) for the PS group while that of PSS was 70.4 (range 62.7–80.2); there was a statistically significant difference (*p* < 0.05) in favor of the group PS. For a more detailed description see Figure 2.

**Figure 3:**
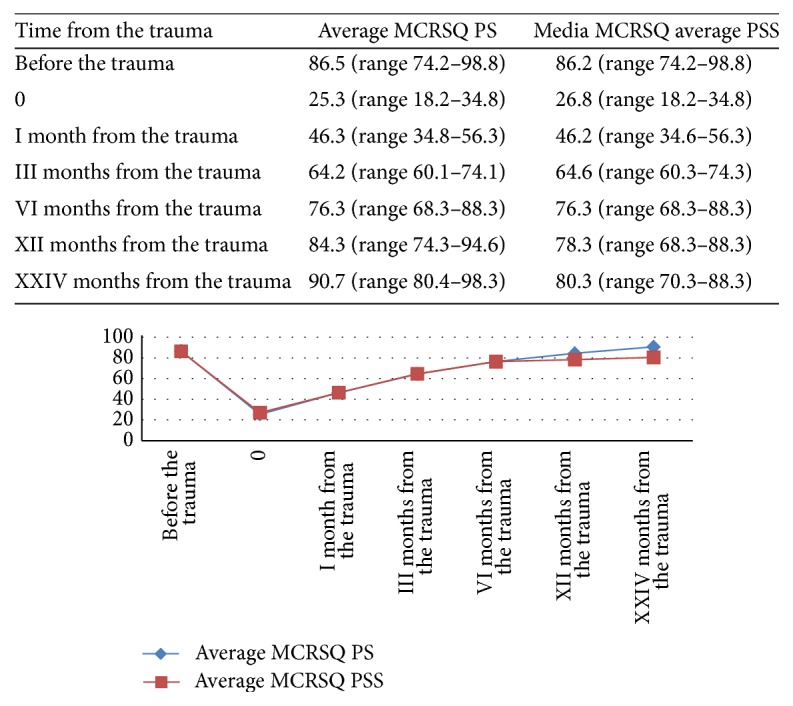
Trend of knee function and quality of life related to it for two-year follow-up measured by the Modified Cincinnati Rating System Questionnaire (MCRSQ). At twelve months there was a statistically significant difference (*p* < 0.05) in favor of the PS group.

MCRSQ before the trauma, for the PS group, was about 86.5 (range 74.2–98.8) points while that of the PSS group was 86.2 (range 74.2–98.8) points. There was no statistically significant difference between the two groups (*p* > 0.5). At the twelfth month, the HHS score was 84.3 (range 74.3–94.6) for the PS group while that of PSS was 78.3 (range 68.3–88.3). There was a statistically significant difference (*p* < 0.05) in favor of the PS group. For a more detailed description see Figure 3.

**Figure 4:**
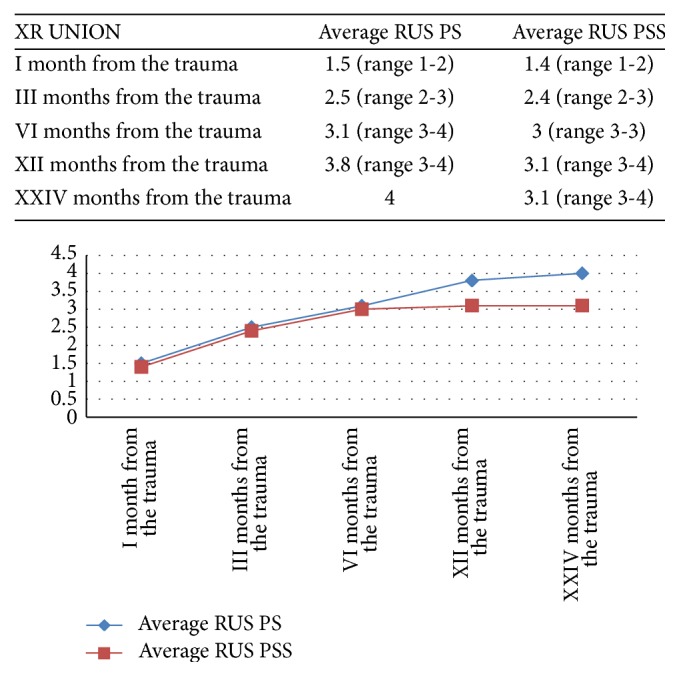
Trend of bone healing in two-year follow-up measured by Radiographic Union Score (RUS). At the twelfth month there was a statistically significant difference (*p* < 0.05) in favor of the PS group.

Regarding the trend of bone healing on a 1-year follow-up measured by RUS, in the twelfth month, there was a statistically significant difference (*p* < 0.05) in favor of the PS group (Figure 4). Bone healing occurred in the PS group on average of 9.6 months after surgery while in the PSS group bone healing occurred 12.4 months postoperatively (Figure 4).

The patients had an indication to the progressive load on average 50.6 days after surgery.

We have not lost any patients from the two groups of up to 24 months of follow-up.

In the PS group, there were 6 complications; the two most frequent were cardiac decompensation (25%, *n* = 2) and myocardial infarction (25%, *n* = 2); after a 2-year follow-up there were 2 deaths, 25% of the total population (Table 3).

In the PSS group there were 12 complications, the most frequent was cardiac decompensation (42.86%, *n* = 3); after a 2-year follow-up there were 2 deaths, 28.57% of the total population (Table 3).

There was no statistically significant difference between the two groups (*p* > 0.5) for postoperative mortality.

## 5. Discussion

More than 80,000 hip replacement operations are performed in Italy every year: a procedure that represents one of the major achievements of modern orthopedic surgery. The complexity of the factors influencing the success of the operations in clinical terms does not facilitate the use of outcome indicators [17]. Current expectations of success have thus made it possible to extend the range to pathologies and age groups initially considered at high risk. Many prospective studies report as future projections an increase of periprosthetic femur and knee fractures. Gathering information on the patients' quality of life in clinical trials of high methodological level is the basis of progress in prosthetic hip and knee surgery. It is known, however, that the most recent and sophisticated clinical trial methodologies emphasize the need for an assessment of a rigorous and standardized outcome [18]. Clinical research in prosthetic hip and knee surgery has focused, in recent years, on the analysis of the results, in order to reveal common features of the various methods in terms of benefits and complications, but also the differences of specific centers and institutions [19]. Similar analysis conducted in different geographical areas (USA, France) has shown how a measuring index of the great diffusion as the Harris Hip Score (HHS), until now considered an undisputed standard and only recently statistically validated [20], actually provides a partial evaluation of the patient's perspective on the pathology and the surgical procedure: only a share of the major causes of the patient's complaints and disability are examined by the questionnaire, while it ignores other complaints (night pain, sexual activity, sleep disorders, etc.) which have a high subjective meaning [13]. In applying a similar procedure to the prosthetic hip and knee surgery, many authors agree to use a combined generic questionnaire (SF-36) [14, 15] for the correct determination to evaluate the quality of life of patients subject to revision of prosthetic implants. In the evaluation of the prosthetic hip surgery a significant problem is the presence of “comorbidity,” that is, the role played by the associated pathologies, especially in the elderly. If we consider that the majority of implants has high survival rates (above 90%) 10 years after surgery and that, to date, in Italy the average age of a patient undergoing this type of operation is of about 70 years, we can easily imagine how associated musculoskeletal, but also cardiovascular, respiratory, and neurological, pathologies produce a continuous decay of functional indexes, which affect the result regardless of the hip or knee prosthesis [16]. This “problem” influences the general measures, as the femoral periprothesic fractures, as recently documented by Ritter and Albohm [21]. The collection of a comorbidity index may facilitate, in the analysis phase, the stratification of patients and has been highly recommended by several authors. Not surprisingly it was Sir John Charnley, father of the modern prosthetic hip surgery and careful scholar of the results of the method he perfected, that designed a simple system to differentiate patients with monoarticular (class A), bilateral (class B) disease or suffering from other chronic diseases (class C), which proves to be still very useful in longitudinal studies [22]. From the surgical point of view in the majority of cases of type B Vancouver fracture (60–75%) [23], the stem may appear mobilized (B2). A surgical alternative to the methods presented by us in these cases is to replace the femoral stem using long stem uncemented prosthesis which is able to exceed the fracture, twice the cortical diameter [24], so as to obtain a good stability, similar to that obtained with an intramedullary nail. Many authors recommend the use of uncemented long stems with a distal porous coating because the use of cement in the fracture can lead to nonhealing and, for the interposition in the fracture, also general risks such as air embolism and vascular problems due to the exothermic reaction during polymerization [25].

In the Vancouver C type fractures (fracture distal to the femoral stem) [26], the femoral stem is generally stable and therefore these injuries can be treated by applying the general concepts of reduction and osteosynthesis, typical for common femoral fracture. It is important to obtain a good anatomic axis reduction and ensure a good stability. To achieve these results, in our opinion, it is preferable to reduce the fracture with ORIF “open” technique and stabilize it with a plate that allows the simultaneous use of screws and/or cerclage. In situations where the subprosthetic fracture is very distal to the stem, to affect the pars metaphyseal distal femur, we can consider a “closed” reduction and stabilization with an intramedullary retrograde nail [26]. The same principle of retrograde nailing in Type 2 or Type 3 Rorabeck is considered by many authors to be a simple, safe, and minimally invasive treatment of femoral periprosthetic knee fractures [26], with high success rates related to the ability of the system to ensure a good axial, angular, and rotational stability which allows early mobilization of the prosthetic knee. Retrograde nailing is not recommended in the presence of very distal and comminuted femur fractures in which the space for the insertion of the distal locking screws may be insufficient. The nailing is also not recommended in the presence of ipsilateral hip replacement since it creates a less resistant area between the stems of the two implants such as causing a fracture in the free interposed zone. The osteosynthesis with plate is primarily recommended in comminuted fractures of the more distal portion of the femur on stable implants (Type 2 Rorabeck), where the construct with multiple converging screws with angular stability provides mechanical stability to the axial and torsional forces acting on bones often osteoporotic [27]. The development of plates with polyaxial screws also allows insertion of screws around any prosthetic implant. Many authors consider useful, especially in highly comminuted fractures, the use of such plates with angular stability with bridge fittings, bypassing the fracture, as long as a correct axial alignment is guaranteed [27]. When possible, the use of LISS plate (Less Invasive Stabilization System) with minimally invasive MIPO technique (Minimally Invasive Plate Osteosynthesis) allows a minimal dissection of the soft tissue and periosteum, reduced blood loss, and reduced risk of infection [27]. The plate osteosynthesis is especially useful in the presence of an implant in the proximal femur (prosthetic rod or pertrochanteric nail) being equipped with monocortical stability screws that permit overlap pin of the distal portion of the implant, so as to avoid an increase of stress between two implants. Ultimately a stable internal fixation with a reduced damage to soft tissues allows an early functional recovery [27]. The use of the plate and the graft of allogenic femoral cortical splint in the treatment of periprosthetic femoral fractures is not a new technique and it has been extensively described by many authors [28]. J.-W. Wang and C.-J. Wang [29] in 2002 recommended the use of a compression plate opposed to cortical splint. Wang's group reported a fracture consolidation rate of 100%, but they also reported a case of osteomyelitis and one malunion after a maximum follow-up of 68 months [29]. From 1996 to 2007, Font-Vizcarra et al. [11] in their study reported a retrospective review of 21 patients who had periprosthetic femoral fractures and were treated with plate and screws instead of allogenic frozen cortical stick. The group was made up of 16 women and 5 men with an average age 80.3 years at time of surgery. Three patients were not available at follow-up and four died within a few weeks after discharge. The remaining 14 patients were evaluated clinically and radiologically with a mean follow-up of 3.2 years. The consolidation of the fracture was observed in 13 patients, and the integration of the transplant occurred in 12 patients. One of the 14 patients developed a deep infection with staphylococcus coagulase-negative, with a satisfying result after surgical debridement and antibiotic therapy. There were no cases of nonunion or implant failure. At final evaluation, the mean EQ-5D VAS score was 64 (ranging from 40 to 90 points) and the EQ-5D mean health index adapted to the Spanish population was 0.57. We used the SF-36's overall score to measure the patients quality of life because we want to understand: evaluating individual patients health status; researching the cost-effectiveness of a treatment; monitoring and comparing disease burden. We are also aware that it has the following limitations: the survey does not take into consideration a sleep variable; the survey has a low response rate in the >65 population.

The mean Oxford hip score was 31.2. The results described by Font-Vizcarra et al. [11] support the use of cortical allograft for these fractures to increase the chance of fracture healing and improve bone biomechanics. The same authors believed that the cortical splint opposed to plate and screws is particularly suitable for the B1 and C fractures in which decreased bone density is present [11].

From the qualitative point of view of the hip function and the quality of life, the patients were given two questionnaires: the HHS and the SF-12 (the simplified form of SF-36). As shown in the new study by Dettoni et al. [30], the validations to adaptation of the Italian population to the HHS, HHS, and SF-12 are correlated with each other. The same correlation can be taken between SF-36 and MCRSQ [31]. All scientific literature agrees that the simple fracture of the proximal femur in an elderly patient with many comorbidities can result, one year after the trauma, in death, entrapment, significant reduction in quality of life, and loss of autonomy in normal activities of normal life [32]. The literature has demonstrated that both morbidity and mortality in the patients suffering from periprosthetic fracture are similar to those of the geriatric hip fracture population. As such, the early restoration of function and ambulation is critical in patients with these injuries, and effective surgical strategies to achieve these goals are essential [32].

The treatment of periprosthetic fractures is very complex and the results are very variable. The treatment for each case must be individualized. Custom-made prosthesis may be used in places where the prosthesis is well fixed in the femur. The goal is to obtain stable fracture fixation and a secure and well-fixed femoral component in proper alignment which allows for early mobilization of the patient to prevent any complications associated with prolonged recumbency in old age [33].

In 2003, Peters et al. [12] in a cadaveric study compared the stability of the periprosthetic femur fracture using the single metal plate of cortical bone splint opposed to a metal plate and the use of two cortical slats with rings. The cadaveric studies were carried out by loading the weight on one leg and climbing the stairs with a force of 2250 Newtons. Stability and optimum implant resistance by loading on one lower limb were achieved in the group of two cortical bone slats and rings. In climbing stairs the most performing implant was the one with cortical bone splint opposed to the metal plate and screws. The authors concluded that the plate alone was not enough to achieve good biomechanical outcomes for this type of fractures.

When comparing the total femoral allograft with osteosynthesis using plate and screws the use of a cortical graft (splint) and a plate is more rigid than the plate itself but it is not as rigid as using two plates positioned orthogonally [34]. The modulus of elasticity is similar between the cortical allograft and host bone but the allogeneic bone splint reduces stress shielding [34]. The use of allogenic cortical splint has the potential to add stem cells, cancellous bone, and bone matrix [35] and is therefore particularly useful for patients who are known to be commonly affected by osteoporosis [35]. Rates of 89–100% of the consolidation of fractures have been reported by using cortical allografts with or without contraposition of plate for periprosthetic femoral fractures [29], and the addition of allograft cancellous bone may increase the rate of fracture healing [35]. In fact Kim et al. [36] revealed that the 16-year rate of survival of the components was 91% with bone allograft strut and the mean Harris Hip Score was 39 ± 10 points before revision and improved to 86 ± 14 points at 16-year follow-up (*p* = 0.02) and the mean preoperative WOMAC score was 62 ± 29 (41–91) points and improved to 22 ± 19 (11–51) points at 16-year follow-up (*p* = 0.003). Fractures treated with plate fixation are more rigid and do not form the same robust callus as those treated with intramedullary nails [37]. Furthermore, the presence of the plate with or without the bone allograft strut obscures the evaluation of the lateral cortex, and many reviewers commented on the difficulty in its scoring. This difficulty is reported in experimental work, where the intraclass correlation coefficient (between RUST and modified RUST) values of individual cortices that demonstrated the lowest agreement for the lateral cortex in plate fixation are seen. The goal of the modified RUST or RUS score described in many articles is to gain a greater range of scores during the crucial time of healing when callus was bridging [37].

The cortical allografts allow personalized fixation without the expense of having a custom-made implant. The use of the slats makes prosthetic revision surgery longer than using the plate alone. The increasing complexity of the most extensive surgery for dissection of the soft tissue and periosteal detachment [38] may explain a high rate of complications; the authors show a 17% of complications [38] and the deep infection has a rate of 4−13% [38]. The great mass of dead bone can increase the rate of deep infection the same way a devitalized bone produces the growth of bacteria leading to infection. The transmission of infectious diseases is possible with the use of allogeneic transplant, but the protocols in use in bone banks have reduced this risk [38].

Complete resorption of the cortical bone splint has rarely been reported in practice; however, the stages that precede this resorption have been observed [38]. In our case history the most recent X-ray examination has shown the melting, but not the reabsorption, and this indicates a cortical graft revascularization [38], which was especially evident in the revisions of knee periprosthetic fractures [38].

## 6. Conclusions

From the data available in the literature and from our experience we can say that the use of the metal plate opposed to the cortical allogenic splint should be considered as a noteworthy point for periprosthetic femur fractures in the hip and knee arthroplasty, where there is bone loss and/or a potential mechanical instability. We have shown that the potential benefit from the association of a metal plate with cortical allogenic splint increases bone quality, reduces stress shielding, increases the percentage of probability of fracture consolidation, makes the system more stable, reduces complications, and improves patient quality of life due to a shorter functional recovery. However the customization of the transplant must be considered against the potential disadvantages of the lengthening of surgical time and the complexity of the surgery, the risk of infections, the nonunion, mortality, and transmission of infectious diseases.

## Figures and Tables

**Table 1:** Description of population.

	PS	PSS
Number of patients	8	7

Average of age in years	77.8	75.3

Range of patients age in years	70–89	67–81

Gender ratio (m : f)	0.6 (3 : 5)	0.75 (3 : 4)

Fractures type according to Vancouver and Rorabeck classification	Vancouver B2: 2 Vancouver C: 3 Rorabeck 2: 2 Rorabeck 3: 1	Vancouver B2: 2 Vancouver C: 3 Rorabeck 2: 1 Rorabeck 3: 1

ASA physical status classification system	ASA I: 0 ASA II: 2 ASA III: 6 ASA IV: 0	ASA I: 0 ASA II: 3 ASA III: 4 ASA IV: 0

Number of patients that needed a place in intensive care	5 (62.5%)	5 (71.42%)

Patients treated for osteoporosis	4 (50%)	6 (85.71%)

Average years of follow-up after periprosthetic fracture	2.3	2.3

Range of years of follow-up after periprosthetic fracture	1–4	1–5

**Table 2:** Patients' comorbidity.

	PS (%)	PSS number (%)
Comorbidity		
Cardiovascular diseases	6 (75%)	5 (71.42%)
Stroke	4 (50%)	1 (14.29%)
Respiratory diseases	5 (62.5%)	6 (85.71%)
Nefro-urologic diseases	5 (62.5%)	2 (28.57%)
Diabetes mellitus	4 (50%)	6 (85.71%)
Rheumatic diseases	4 (50%)	7 (100%)
Parkinson's disease	1 (12.5%)	2 (28.57%)
Smokers	1 (12.5%)	3 (42.86%)
Use of steroids	7 (87.5%)	7 (100%)
Number of comorbidities for patient		
1	1 (12.5%)	1 (14.29%)
2	2 (25%)	1 (14.29%)
≥3	5 (62.5%)	5 (71.42%)

**Table 3:** Postoperative complications during all the follow-up.

	PS (%)	PSS (%)
Respiratory infections	1 (12.5%)	1 (14.29%)
Cardiac failure	2 (25%)	3 (42.86%)
DVE (Deep Venous Thrombosis)	0 (0%)	0 (0%)
Urinary infection	0 (0%)	2 (28.57%)
Gastrointestinal bleeding	0 (0%)	2 (28.57%)
Myocardial infarction	2 (25%)	2 (28.57%)
Ictus/tia	1 (12.5%)	2 (28.57%)
Number of complications for patient:		
1	1 (12.5%)	1 (14.29%)
2	1 (12.5%)	2 (28.57%)
≥3	1 (12.5%)	2 (28.57%)
Numbers of deaths		
After two years of follow-up	2 (25%)	2 (28.57%)
